# Integration of hospital with congregate care homes in response to the COVID-19 pandemic

**DOI:** 10.14745/ccdr.v49i23a06

**Published:** 2023-02-01

**Authors:** Christina K Chan, Mercedes Magaz, Victoria R Williams, Julie Wong, Monica Klein-Nouri, Sid Feldman, Jaclyn O’Brien, Natasha Salt, Andrew E Simor, Jocelyn Charles, Brian M Wong, Steve Shadowitz, Karen Fleming, Adrienne K Chan, Jerome A Leis

**Affiliations:** 1 Infection Prevention and Control, Sunnybrook Health Sciences Centre, Toronto, ON; 2 Centre for Quality Improvement and Patient Safety, Temerty Faculty of Medicine, University of Toronto, Toronto, ON; 3 Meighen Health Centre, Toronto, ON; 4 Apotex Centre, Jewish Home for the Aged, Baycrest Health Sciences, Toronto, ON; 5 Division of Infectious Diseases, Department of Medicine, Sunnybrook Health Sciences Centre, Toronto, ON; 6 Veterans Centre, Sunnybrook Health Sciences Centre, Toronto, ON; 7 Department of Family and Community Medicine, Sunnybrook Health Sciences Centre, Toronto, ON; 8 Division of General Internal Medicine, Department of Medicine, Sunnybrook Health Sciences Centre, Toronto, ON

**Keywords:** COVID-19, SARS-CoV-2, long-term care, infection prevention and control, IPAC, congregate care, retirement homes

## Abstract

**Background:**

The coronavirus disease 2019 (COVID-19) pandemic has highlighted the need to improve the safety of the environments where we care for older adults in Canada. After providing assistance during the first wave, many Ontario hospitals formally partnered with local congregate care homes in a “hub and spoke” model during second pandemic wave onward. The objective of this article is to describe the implementation and longitudinal outcomes of residents in one hub and spoke model composed of a hospital partnered with 18 congregate care homes including four long-term care and 14 retirement or other congregate care homes.

**Intervention:**

Homes were provided continuous seven-day per week access to hospital support, including infection prevention and control (IPAC), testing, vaccine delivery and clinical support as needed. Any COVID-19 exposure or transmission triggered a same-day meeting to implement initial control measures. A minimum of weekly on-site visits occurred for long-term care homes and biweekly for other congregate care homes, with up to daily on-site presence during outbreaks.

**Outcomes:**

Case detection among residents increased following implementation in context of increased testing, then decreased post-immunization until the Omicron wave when it peaked. After adjusting for the correlation within homes, COVID-related mortality decreased following implementation (OR=0.51, 95% CI, 0.30–0.88; *p*=0.01). In secondary analysis, homes without pre-existing IPAC programs had higher baseline COVID-related mortality rate (OR=19.19, 95% CI, 4.66–79.02; *p*<0.001) and saw a larger overall decrease during implementation (3.76% to 0.37%–0.98%) as compared to homes with pre-existing IPAC programs (0.21% to 0.57%–0.90%).

**Conclusion:**

The outcomes for older adults residing in congregate care homes improved steadily throughout the COVID-19 pandemic. While this finding is multifactorial, integration with a local hospital partner supported key interventions known to protect residents.

## Introduction

Individuals who reside in congregate care (CC) homes, including long-term care (LTC) and retirement homes (RH), have been disproportionately affected by the coronavirus disease 2019 (COVID-19) pandemic in Canada ((1–3)). During the first wave of the COVID-19 pandemic, a greater proportion of COVID-19 deaths in Canada occurred in LTCs as compared to other countries in the Organisation for Economic Co-operation and Development ((2)).

Multiple system gaps were identified as contributing to extensive COVID-19 transmission in these homes, including lack of formal infection prevention and control (IPAC) programs and insufficient human and physical resources for resident care ((4–7)). A prior description of one of the first outbreaks of COVID-19 in Canada demonstrated how undetected rapid spread of severe acute respiratory syndrome coronavirus 2 (SARS-CoV-2) occurred in homes that lacked surveillance and control measures ((7)).

In October 2020, Ontario Health, a government agency charged with connecting and coordinating the province’s healthcare system, established a “hub and spoke” model where some hospitals were formally partnered with their local community CC homes to support IPAC ((8)). The objective of this study was to describe the implementation and longitudinal outcomes of residents in one hub and spoke program in Toronto, Canada.

## Intervention

Sunnybrook Health Sciences Centre is an academic health sciences centre in Toronto that began to support on-site management of COVID-19 outbreaks in local CC homes as early as April 2020. The formalized hub and spoke model was funded by Ontario Health and officially launched on October 6, 2020, across north Toronto following a webinar between all partner organizations outlining expectations and available resources. The north Toronto hub team was composed of a 0.8 full-time equivalent (FTE) IPAC medical director, 1.0 FTE IPAC operations lead, 1.0 FTE IPAC coordinator per 280 LTC beds and 1.0 FTE IPAC coordinator per 600 RH beds. This team was integrated with the existing manager of strategy and integration for the hospital, and physicians from family medicine, general internal medicine and infectious disease as needed. The ”spokes” consisted of 18 CC homes including 4 LTC (1,116 beds) and 14 RH or other CC homes (1,543 beds). There were 16 (88.9%) facilities with exclusively private rooms and nine (50.0%) that were for-profit organizations. Each home’s leadership and internally appointed IPAC lead worked directly with the hub daily. Three facilities (two LTC, one RH) already had structured IPAC programs at baseline meaning that they had dedicated on-site IPAC personnel before the creation of the hub.

The intervention involved continuous seven-day per week access to the hospital hub to support IPAC, diagnostic testing and vaccine delivery and administration as needed. A secure group email was created to reach the hub, which was monitored continuously by hub members to ensure timely support. On-site visits occurred minimum weekly for LTC and biweekly for other CC homes, with up to daily on-site presence during outbreak periods. Active surveillance was performed minimum daily at each site and access to nasopharyngeal testing for SARS-CoV-2 polymerase chain reaction (PCR) testing was supported by the hospital hub as needed. Any confirmed case of COVID-19 among residents or exposure by staff who worked during their period of infectivity was reported to the hub and triggered a same-day virtual meeting to implement control measures and/or on-site visits as required. Support with collection of SARS-CoV-2 testing and clinical management was deployed as needed, including within-home treatment of residents and/or direct transfer to the hospital ward as appropriate.

Iterative improvements to IPAC were made in partnership with homes during site visits, across the hierarchy of hazard controls. Elimination controls focused on vaccination against COVID-19 for all residents and staff starting in December 2020, including supplying and administering the primary series and booster doses. Engineering controls included assessment and optimization of heating, ventilation and air conditioning systems as needed, installation of portable high-efficiency particulate air filters and limiting occupancy of shared rooms where possible. Administrative controls included deployment of standardized signage, and dedicated training of staff regarding personal protective equipment (PPE) use, hand hygiene, environmental cleaning and disinfection and other IPAC practises. Formal audits were performed by hospital hub using a standard tool adapted from the World Health Organization and Public Health Ontario ((9,10)). These audits included five IPAC components scored on a five-point scale each including hand hygiene, environmental cleaning, use of PPE, screening and adherence to physical distancing where appropriate (**Supplemental material, Table S1**).

## Evaluation

A multicentre prospective quality improvement study was conducted comparing five study periods: baseline (wave one; March 1, 2020, to June 30, 2020), implementation period 1 pre-immunization (wave two; October 1, 2020, to December 31, 2020), implementation period 2 post-immunization (waves two and three; January 1, 2021, to May 31, 2021), implementation period 3 post-immunization (wave four; August 1, 2021, to December 14, 2021) and implementation period 4 post-immunization (wave five; December 15, 2021, to February 28, 2022). **Table 1** describes the broader context of each study period in terms of factors influencing the outcome of residents in CC homes, while **Table 2** provides the baseline characteristics of these homes during each period.

**Figure 1:**
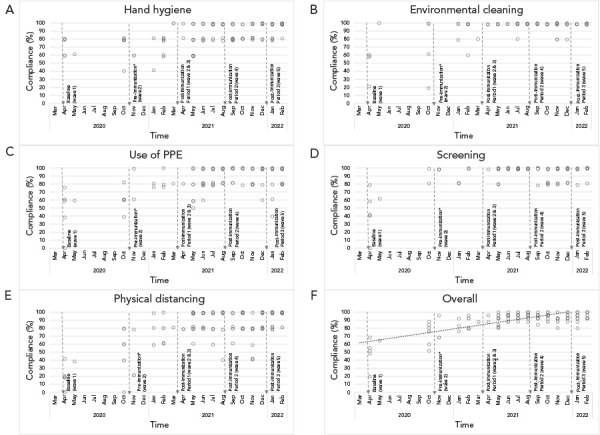
Scatter plots of the compliance with infection prevention and control practises of the 18 congregate care homes before and after implementation of “hub and spoke” program Abbreviation: PPE, personal protective equipment

**Figure 2:**
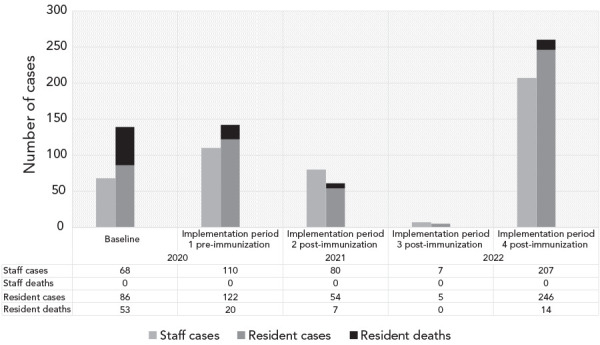
Number of COVID-19 resident and staff cases across 18 congregate care homes following implementation of “hub and spoke” program Abbreviation: COVID-19, coronavirus disease 2019

**Table 1:** Summary of COVID-19 context during the five study periods

***Factors influencing outcomes of residents in congregate care settings***	**Baseline** **(Wave 1 pre-immunization, original virus)**	**Implementation period 1 pre-immunization** **(Wave 2, original virus)**	**Implementation period 2 post-immunization** **(Wave 2 and 3, Alpha variant)**	**Implementation period 3 post-immunization** **(Wave 4, Delta variant)**	**Implementation period 4 post-immunization** **(Wave 5, Omicron variant)**
*Community rate*	Moderate^a^	Moderate^a^	Moderate^a^	Lowest^b^	Highest^c^
*IPAC programs*	None^c,d^	In place^b^	In place^b^	In place^b^	In place^b^
*COVID-19 vaccine*	None^c^	None^c^	Implemented (1–2 doses)^b^	2–3 doses^b^	3–4 doses^b^
*Vaccine effectiveness*	None^c^	None^c^	High ((11)) (63%–82% with 1 dose and 89%–92% with 2 doses)^b^	High ((12)) (87%–95% with 2 doses and 97% with 3 doses)^b^	Reduced ((12)) (61% with 3 doses)^a^
*Vaccine protection against severe outcomes*	None^c^	None^c^	High ((11)) (80%–87% with 1 dose and 82%–96% with 2 doses)^b^	High ((11,12)) (91%–98% with 2 doses and 99% with 3 doses)^b^	High ((12)) (95% with 3 doses)^b^
*Therapeutics available*	None^c^	Dexamethasone^b^	Dexamethasone Tocilizumab^b^	Dexamethasone Tocilizumab Remdesivir^b^	Dexamethasone Tocilizumab Remdesivir Sotrovimab Baricitinib^b^

Abbreviations: COVID-19, coronavirus disease 2019; IPAC, infection prevention and control

^a^ Factors that conferred some protection of residents in congregate care settings

^b^ Factors that conferred significant protection of residents in congregate care settings

^c^ Factors that conferred no protection of residents in congregate care settings

^d^ Three congregate care homes had formal IPAC homes at baseline

**Table 2:** Characteristics of the 18 congregate care homes before and after implementation of “hub and spoke” program supporting response to the COVID-19 pandemic

Characteristics of congregate care homes	Baseline (Wave 1 pre-immunization, original virus)	Implementation period 1 pre-immunization (Wave 2, original virus)	Implementation period 2 post-immunization (Wave 2 and 3, Alpha variant)	Implementation period 3 post-immunization (Wave 4, Delta variant)	Implementation period 4 post-immunization (Wave 5, Omicron variant)
**Staff**					
Number of staff	2,389	2,259	2,208	2,454	2,632
Average number of staff in LTC	350	333	325	372	434
Average number of staff in RH	71	66	65	69	64
Staff vaccinated with 1 dose only, n (%)	N/A	N/A	494 (21.9)	418 (17.0)	3 (0.1)
Staff vaccinated with 2 doses only, n (%)	N/A	N/A	1,369 (58.5)	1,908 (77.8)	1,369 (51.7)
Staff vaccinated with 3 doses, n (%)	N/A	N/A	N/A	N/A	1,257 (47.5)
**Residents**					
Number of residents	2,325	2,134	2,043	2,108	2,231
LTC	1,034	912	852	890	931
RH	1,291	1,222	1,191	1,218	1,300
Age of residents, years, mean (SD)	84.5 (8.4)	85.1 (7.8)	84.9 (7.7)	83.8 (7.9)	86.6 (5.5)
Female residents, n (%)	1,408 (60.8)	1,291 (60.6)	1,274 (57.7)	1,251 (59.3)	1,311 (58.8)
Residents vaccinated with 1 dose only, n (%)	N/A	N/A	31 (1.5)	92 (4.4)	22 (1.1)
Residents vaccinated with 2 doses only, n (%)	N/A	N/A	1,925 (94.2)	1,910 (90.6)	183 (8.2)
Residents vaccinated with 3 doses, n (%)	N/A	N/A	N/A	N/A	1,981 (88.8)

Abbreviations: COVID-19, coronavirus disease 2019; LTC, long-term care; N/A, not applicable; RH, retirement home; SD, standard deviation

Process measures were prospectively tracked to assess implementation of interventions including number of emails received/sent by the hub, number of on-site visits for IPAC or testing or vaccination, number of town halls/webinars and number of virtual meetings.

The primary outcome was the incidence of COVID-19-related mortality among residents defined as the rate of death due to COVID-19 across the entire home. Attribution of death was based on the home’s physician review and categorization reported to the local public health unit. Secondary outcomes included proportion of residents who developed laboratory-confirmed COVID-19, resident COVID-19 case fatality rate defined as death within 30 days of start of infection, staff COVID-19 infection rate defined as overall infection rate including community-acquired cases, the number of PCR tests performed per day (including testing of both residents and staff), and adherence to IPAC practises based on site audits by hospital hub IPAC specialists. Infection and mortality rates were calculated based on the number of residents residing in the home and the number of employees at the start of each study period. Dichotomous outcomes between different periods were compared using a logistic regression model that adjusted for correlation within homes. Scatter plot diagrams were used to visually compare IPAC practises across the five study periods.

As a secondary analysis, the primary outcome of the three facilities with pre-existing structured IPAC programs were combined as a control group, to assess for any difference compared to the remaining homes, both at baseline and during implementation. Finally, to partially address potential for survivor bias, a sensitivity analysis was performed where the analysis was repeated with the exclusion of residents with COVID-19 during wave one who survived from implementation period one.

Research ethics review to complete this evaluation was not required because the study met criteria for exemption as the project was deemed quality improvement and not human subject research.

## Outcomes

**Table 3** summarizes the process measures of hub and spoke implementation. In total throughout the intervention, there were 4,051/4,142 emails sent/received by the hub, 631 on-site visits, 70 hub and spoke meetings, 9 town halls/webinar, 196 outbreak meetings, 49 vaccine support visits and 27 visits to support nasopharyngeal PCR testing. **Figure 1** depicts a scatter plot of adherence to IPAC practises over time where each point represents an on-site audit. Measurable improvements were observed across all areas, which were generally sustained (see trend line).

**Table 3:** Process measures in the 18 congregate care homes before and after implementation of “hub and spoke” program supporting response to the COVID-19 pandemic

Process measures	Baseline (Wave 1 pre-immunization, original virus)	Implementation period 1 pre-immunization (Wave 2, original virus)	Implementation period 2 post-immunization (Wave 2 and 3, Alpha variant)	Implementation period 3 post-immunization (Wave 4, Delta variant)	Implementation period 4 post-immunization (Wave 5, Omicron variant)
Total number of visits conducted by IPAC	N/A	98	193	209	131
Median weekly visits conducted by IPAC (IQR)	N/A	7 (3.8)	9 (4.0)	11 (2.5)	12 (2.0)
Total number of hub and spoke meetings	N/A	14	23	23	10
Median weekly hub and spoke meetings (IQR)	N/A	1 (0.0)	1 (0.0)	1 (0.5)	1 (0.5)
Total number of town halls/webinars	N/A	6	1	2	0
Total number of outbreak meetings	N/A	50	58	16	72
Median weekly outbreak meetings (IQR)	N/A	4 (6.0)	4 (2.0)	1 (2.0)	6 (7.0)
Median weekly emails received from the homes (IQR)	N/A	27 (26.5)	66 (33.0)	26 (12.5)	154 (165.5)
Median weekly emails sent to the homes (IQR)	N/A	28 (21.5)	77 (37.0)	28 (9.5)	145 (99.0)
Total number of visits for vaccine support	N/A	N/A	24	15	10
Total number of visits for collecting PCR samples	N/A	16	8	0	3

Abbreviations: COVID-19, coronavirus disease 2019; IPAC, infection prevention and control; IQR, interquartile range; N/A, not applicable; PCR, polymerase chain reaction

The primary and secondary outcomes are described in **Table 4** and the results of logistic regression are shown in **Table 5.** **Figure 2** also depicts the total number of residents and staff cases as well as deaths during the study periods. The COVID-19-related mortality in residents decreased in implementation period 1 (OR=0.51, 95% CI, 0.30–0.88; *p*=0.01), which was sustained throughout the implementation periods. Resident case fatality decreased steadily from 38.1% at baseline to a nadir of 0%–5.1% (OR=0.08, 95% CI, 0.03–0.20, *p*<0.001). In the context of increased PCR testing (Table 4), resident case detection increased (OR=1.32, 95% CI, 1.02–1.71, *p*=0.03) during implementation period 1, then decreased post-immunization until the Omicron wave (period 4) when it peaked (OR=2.20, 95% CI, 1.75–2.77, *p*<0.001).

**Table 4:** Resident and staff outcomes in the 18 congregate care homes before and after implementation of ‘”hub and spoke” program supporting response to the COVID-19 pandemic

Outcome measures	Baseline (Wave 1 pre-immunization, original virus)	Implementation period 1 pre-immunization (Wave 2, original virus)	Implementation period 2 post-immunization (Wave 2 and 3, Alpha variant)	Implementation period 3 post-immunization (Wave 4, Delta variant)	Implementation period 4 post-immunization (Wave 5, Omicron variant)
Resident COVID-19-related mortality	2.3%	0.9%	0.3%	0.0%	0.6%
Resident case fatality rate	38.1%	14.1%	11.7%	0.0%	5.1%
Proportion of residents positive for SARS-CoV-2 by PCR^a^	6.0%	6.7%	2.9%	0.1%	11.4%
Percentage of staff positive for SARS-CoV-2 by PCR^a^	2.8%	4.9%	3.0%	0.0%	7.4%
Number of PCR tests for SARS-CoV-2 per 100 residents and staff—total (daily average)	314 (2.6)	5,214 (57.3)	4,048 (27.1)	390 (2.9)	1,875 (25.0)

Abbreviations: COVID-19, coronavirus disease 2019; PCR, polymerase chain reaction; SARS-CoV-2, severe acute respiratory syndrome coronavirus 2

^a^ Overall positivity-rate inclusive of community-acquired cases

**Table 5:** Logistic regression analysis, adjusting for correlation within homes, of outcome measures across 18 congregate care homes before and after implementation of “hub and spoke” program^a^

Outcome measure	OR	95% CI	*p*-value
**COVID-19-related mortality**			
Implementation period 1 pre-immunization (wave 2, original virus)	0.51	0.30–0.88	0.01
Implementation period 2 post-immunization (waves 2 and 3, Alpha variant)	0.18	0.08–0.40	<0.001
Implementation period 3 post-immunization (wave 4, Delta variant)	N/A	N/A	N/A
Implementation period 4 post-immunization (wave 5, Omicron variant)	0.23	0.12–0.43	<0.001
**Case fatality rate**			
Implementation period 1 pre-immunization (wave 2, original virus)	0.52	0.16–1.72	0.28
Implementation period 2 post-immunization (waves 2 and 3, Alpha variant)	0.30	0.08–1.12	0.07
Implementation period 3 post-immunization (wave 4, Delta variant)	N/A	N/A	N/A
Implementation period 4 post-immunization (wave 5, Omicron variant)	0.08	0.03–0.20	<0.001
**Cases detected among residents**			
Implementation period 1 pre-immunization (wave 2, original virus)	1.32	1.02–1.71	0.03
Implementation period 2 post-immunization (waves 2 and 3, Alpha variant)	0.53	0.39–0.73	<0.001
Implementation period 3 post-immunization (wave 4, Delta variant)	0.021	0.01–0.07	<0.001
Implementation period 4 post-immunization (wave 5, Omicron variant)	2.20	1.75–2.77	<0.001

Abbreviations: CI, confidence interval; COVID-19, coronavirus disease 2019; N/A, non-applicable because no resident deaths during implementation period 3; OR, odds ratio

^a^ Baseline (wave 1 pre-COVID-19 immunization, original virus) is the reference category

In secondary analysis, homes without pre-existing IPAC programs had higher baseline COVID-related mortality rate (OR=19.19, 95% CI, 4.66–79.02; *p*<0.001) and saw a larger overall decrease during implementation (3.76% to 0.37%–0.98%) as compared to homes with pre-existing IPAC programs (0.21% to 0.57%–0.90%) (Supplemental material, **Table S2**). In the sensitivity analysis, the reduction in COVID-related mortality among all CC homes remained significant following implementation of the intervention (Supplemental material, **Table S3**).

## Discussion

In the present prospective study, the outcomes of residents across LTC, RH and other CC homes across northern Toronto improved steadily following wave one of the COVID-19 pandemic. Multiple factors likely contributed to better resident outcomes. Prior to COVID-19 vaccination being available in late 2020, these factors likely included earlier detection of cases through surveillance and testing, earlier initiation of supportive therapy, better coordination for those requiring transfer to hospital and increased human resources to support care needs. In addition, dexamethasone and tocilizumab utilization and changes in mechanical ventilation strategies for those transferred to hospital during the second wave onward were recognized interventions that led to improved outcomes of the most severe forms of COVID-19 ((13–15)). In the post-immunization period, resident outcomes improved further owing to high vaccine uptake including timely booster doses, along with the apparent association of the Omicron variant causing less severe disease, as well as broader access to therapeutics.

The implementation of the hub and spoke program helped to support many of these interventions, including adherence to IPAC practises, clinical management and vaccine delivery, and in doing so may have contributed to improved outcomes. Our program implementation was similar to others in both Canada and the United States that contributed to improved resident outcomes in LTC subsequent to the first wave of the COVID-19 pandemic ((16–19)). In Seattle, Washington, where the first reported LTC outbreak occurred in early 2020, a health system response was implemented that included improved communication around the status of LTC homes, early testing and isolation when COVID-19 cases were suspected in the home, and deployment of an on-site team in the case of a COVID-19 outbreak ((19)). One key difference with our hub and spoke model is that our team was on-site even in the absence of COVID-19 activity, working in partnership to strengthen IPAC in anticipation of future pandemic waves. We prospectively measured quantitative improvements in various IPAC practises over time.

A number of important insights arose during implementation of this model of care. First, the weekly meetings and on-site visits created a strong partnership that resulted in improved coordination at multiple levels. For example, surveillance and testing were facilitated resulting in improved turnaround times from specimen collection to result reporting. These newly detected cases were managed in real-time and residents with acute illness were identified based on early warning signs, and in many instances, we were able to facilitate transfers to the hospital directly to an inpatient unit while bypassing the emergency department. Second, the use of virtual platforms allowed teams from multiple institutions to meet seamlessly across different physical locations and to provide consultative services to residents and families in their home. At the same time, we found that virtual care was not a substitute to going on-site to assess IPAC practises and residents in-person on a regular basis. One of our program successes was the on-site presence that is crucial to supporting implementation within the workflow of the home. Third, the adoption of this model resulted in better coordination of resources compared to each CC home navigating the COVID-19 pandemic on its own. For example, the improved visibility around the IPAC status of each home in north Toronto allowed for both hospital and other community care agency resources to be deployed to homes in response to their needs, which prevented critical shortages in human resources and supplies that were seen during the first wave of the COVID-19 pandemic ((4,7)).

### Limitations

Our study has several important limitations. First, it is an observational study describing program implementation and the resident outcomes may be influenced by other confounding factors including the changing context of the pandemic outlined in Table 1. However, many of these protective measures were facilitated through the hub and spoke model. In addition, similar improvements were not observed in the homes with pre-existing IPAC programs, suggesting that the IPAC capacity-building contributed to the improved outcomes among homes that lacked formal IPAC programs at the start of the pandemic. Second, we cannot fully exclude the role of survivor bias leading to improved outcomes in these homes following wave one of the pandemic; however, a sensitivity analysis, which at least partially adjusted for this, still found a significant improvement in resident outcomes following implementation of the intervention. Finally, this evaluation focused only on one hub and spoke intervention implemented in Ontario, Canada, and implementation may have varied elsewhere. Nevertheless, our evaluation provides lessons learned regarding successful implementation of this model.

## Conclusion

The outcomes of older adults residing in CC homes steadily improved throughout the first two years of the COVID-19 pandemic. While this finding is multifactorial, integration with the local hospital partner supported key interventions known to protect residents. Further longitudinal support in IPAC is needed beyond the COVID-19 pandemic to improve the safety of CC environments in Canada.

## Supplemental material

These documents can be accessed on the Supplemental material file.
Table S1: Infection prevention and control audit tool for congregate care homes
Table S2: Resident COVID-19-related mortality in congregate care homes with or without pre-existing infection prevention and control program before the pandemic
Table S3: Sensitivity analysis comparing baseline versus implementation period 1 with COVID-19 recovered residents excluded

## References

[1] Liu M, Maxwell CJ, Armstrong P, Schwandt M, Moser A, McGregor MJ, Bronskill SE, Dhalla IA. COVID-19 in long-term care homes in Ontario and British Columbia. CMAJ 2020;192(47):E1540–6. 10.1503/cmaj.201860 32998943 PMC7721263

[2] Canadian Institute for Health Information. Pandemic experience in the long-term care sector: How does Canada compare with other countries? Ottawa, ON: CIHI; 2020. https://www.cihi.ca/sites/default/files/document/covid-19-rapid-response-long-term-care-snapshot-en.pdf

[3] Costa AP, Manis DR, Jones A, Stall NM, Brown KA, Boscart V, Castellino A, Heckman GA, Hillmer MP, Ma C, Pham P, Rais S, Sinha SK, Poss JW. Risk factors for outbreaks of SARS-CoV-2 infection at retirement homes in Ontario, Canada: a population-level cohort study. CMAJ 2021;193(19):E672–80. 10.1503/cmaj.202756 33972220 PMC8158001

[4] Government of Ontario. Ministry of Long-Term Care. Ontario’s Long-Term Care COVID-19 Commission: final report and progress on interim recommendations. Toronto, ON: MLTC; 2021. [Accessed 2022 Aug 20]. https://www.ontario.ca/page/long-term-care-covid-19-commission-progress-interim-recommendations

[5] Brown KA, Jones A, Daneman N, Chan AK, Schwartz KL, Garber GE, Costa AP, Stall NM. Association between nursing home crowding and COVID-19 infection and mortality in Ontario, Canada. JAMA Intern Med 2021;181(2):229–36. 10.1001/jamainternmed.2020.6466 33165560 PMC7653540

[6] Stall NM, Jones A, Brown KA, Rochon PA, Costa AP. For-profit long-term care homes and the risk of COVID-19 outbreaks and resident deaths. CMAJ 2020;192(33):E946–55. 10.1503/cmaj.201197 32699006 PMC7828970

[7] Murti M, Goetz M, Saunders A, Sunil V, Guthrie JL, Eshaghi A, Zittermann S, Teatero S, Fittipaldi N, Rilkoff H, Gubbay JB, Garber G, Callery S, Holt AM, Noseworthy AL. Investigation of a severe SARS-CoV-2 outbreak in a long-term care home early in the pandemic. CMAJ 2021;193(19):E681–8. 10.1503/cmaj.202485 33972221 PMC8158000

[8] Government of Ontario. Ministry of Health. Infection Prevention and Control Hubs. Toronto, ON: OMH; 2021. [Accessed 2022 Aug 20]. https://www.health.gov.on.ca/en/pro/programs/publichealth/coronavirus/docs/2019_guidance_ipac.pdf

[9] Government of Ontario. Public Health Ontario. COVID-19: Self-Assessment Audit Tool for Long-term Care Homes and Retirement Homes. Toronto, ON: PHO; 2022. [Accessed 2022 Aug 31]. https://www.publichealthontario.ca/-/media/documents/ncov/ltcrh/2021/12/covid-self-assessment-audit-tool-ltc.pdf?sc_lang=en%22

[10] World Health Organization. Infection prevention and control health-care facility response for COVID-19: A module from the suite of health service capacity assessments in the context of the COVID-19 pandemic. Geneva (CH): WHO; 2020. [Accessed 2022 Aug 31]. https://hlh.who.int/docs/librariesprovider4/data-monitoring/infection-prevention-and-control-health-care-facility-response-for-covid-19.pdf

[11] Nasreen S, Chung H, He S, Brown KA, Gubbay JB, Buchan SA, Fell DB, Austin PC, Schwartz KL, Sundaram ME, Calzavara A, Chen B, Tadrous M, Wilson K, Wilson SE, Kwong JC; Canadian Immunization Research Network (CIRN) Provincial Collaborative Network (PCN) Investigators. Effectiveness of COVID-19 vaccines against symptomatic SARS-CoV-2 infection and severe outcomes with variants of concern in Ontario. Nat Microbiol 2022;7(3):379–85. 10.1038/s41564-021-01053-0 35132198

[12] Buchan SA, Chung H, Brown KA, Austin PC, Fell DB, Gubbay JB, Nasreen S, Schwartz KL, Sundaram ME, Tadrous M, Wilson K, Wilson SE, Kwong JC; Canadian Immunization Research Network (CIRN) Provincial Collaborative Network (PCN) Investigators. Estimated Effectiveness of COVID-19 Vaccines Against Omicron or Delta Symptomatic Infection and Severe Outcomes. JAMA Netw Open 2022;5(9):e2232760. 10.1001/jamanetworkopen.2022.32760 36136332 PMC9500552

[13] Horby P, Lim WS, Emberson JR, Mafham M, Bell JL, Linsell L, Staplin N, Brightling C, Ustianowski A, Elmahi E, Prudon B, Green C, Felton T, Chadwick D, Rege K, Fegan C, Chappell LC, Faust SN, Jaki T, Jeffery K, Montgomery A, Rowan K, Juszczak E, Baillie JK, Haynes R, Landray MJ; RECOVERY Collaborative Group. Dexamethasone in Hospitalized Patients with Covid-19. N Engl J Med 2021;384(8):693–704. 10.1056/NEJMoa2021436 32678530 PMC7383595

[14] Gordon AC, Mouncey PR, Al-Beidh F, Rowan KM, Nichol AD, Arabi YM, Annane D, Beane A, van Bentum-Puijk W, Berry LR, Bhimani Z, Bonten MJ, Bradbury CA, Brunkhorst FM, Buzgau A, Cheng AC, Detry MA, Duffy EJ, Estcourt LJ, Fitzgerald M, Goossens H, Haniffa R, Higgins AM, Hills TE, Horvat CM, Lamontagne F, Lawler PR, Leavis HL, Linstrum KM, Litton E, Lorenzi E, Marshall JC, Mayr FB, McAuley DF, McGlothlin A, McGuinness SP, McVerry BJ, Montgomery SK, Morpeth SC, Murthy S, Orr K, Parke RL, Parker JC, Patanwala AE, Pettilä V, Rademaker E, Santos MS, Saunders CT, Seymour CW, Shankar-Hari M, Sligl WI, Turgeon AF, Turner AM, van de Veerdonk FL, Zarychanski R, Green C, Lewis RJ, Angus DC, McArthur CJ, Berry S, Webb SA, Derde LP; REMAP-CAP Investigators. Interleukin-6 Receptor Antagonists in Critically Ill Patients with Covid-19. N Engl J Med 2021;384(16):1491–502. 10.1056/NEJMoa2100433 33631065 PMC7953461

[15] Behesht Aeen F, Pakzad R, Goudarzi Rad M, Abdi F, Zaheri F, Mirzadeh N. Effect of prone position on respiratory parameters, intubation and death rate in COVID-19 patients: systematic review and meta-analysis. Sci Rep 2021;11(1):14407. 10.1038/s41598-021-93739-y 34257366 PMC8277853

[16] Stall NM, Farquharson C, Fan-Lun C, Wiesenfeld L, Loftus CA, Kain D, Johnstone J, McCreight L, Goldman RD, Mahtani R. A hospital partnership with a nursing home experiencing a COVID-19 outbreak: description of a multiphase emergency response in Toronto, Canada. J Am Geriatr Soc 2020;68(7):1376–81. 10.1111/jgs.16625 32441770 PMC7280605

[17] Archbald-Pannone LR, Harris DA, Albero K, Steele RL, Pannone AF, Mutter JB. COVID-19 collaborative model for an academic hospital and long-term care facilities. J Am Med Dir Assoc 2020;21(7):939–42. 10.1016/j.jamda.2020.05.044 32563752 PMC7247468

[18] Lamb MJ, La Delfa A, Sawhney M, Adams D, Abdel-Shahied K, Belfer T, Schembri J, Katz K. Implementation and evaluation of an IPAC SWAT team mobilized to long-term care and retirement homes during the COVID-19 pandemic: A pragmatic health system innovation. J Am Med Dir Assoc 2021;22(2):253–255.e1. 10.1016/j.jamda.2020.11.033 33406385 PMC7833812

[19] Kim G, Wang M, Pan H, H Davidson G, Roxby AC, Neukirch J, Lei D, Hawken-Dennis E, Simpson L, D Ong T. A Health System Response to COVID-19 in Long-Term Care and Post-Acute Care: A Three-Phase Approach. J Am Geriatr Soc 2020;68(6):1155–61. 10.1111/jgs.16513 32343363 PMC7267583

